# Soft Tissue Applications of Er,Cr:YSGG Laser in Pediatric Dentistry

**DOI:** 10.5005/jp-journals-10005-1432

**Published:** 2017-06-01

**Authors:** Gyanendra Kumar, Ferah Rehman, Vivek Chaturvedy

**Affiliations:** 1 Research Associate, Department of Pedodontics and Preventive Dentistry, Maulana Azad Institute of Dental Sciences, New Delhi, India; 2 Associate Professor, Department of Pedodontics and Preventive Dentistry, Maulana Azad Institute of Dental Sciences, New Delhi, India; 3 Professor and Principal, Department of Pedodontics and Preventive Dentistry, Eklavya Dental College, Kothputli, Rajasthan, India

**Keywords:** Frenectomy, Mucocoele, Pyogenic granuloma.

## Abstract

Soft tissue laser, such as diode and Nd:YAG lasers were initially used in soft tissue lesions because of its increased success rate. It was because of the fact that these lasers were well-absorbed by chromophores, such as hemoglobin and melanin which are found abundant in the oral mucosa. The introduction of erbium family in 1990 comprising the Er:YAG and Er,Cr:YSGG lasers made the hard tissue laser a boon for dentistry. Erbium, chromium-doped yttrium, scandium, gallium and garnet (Er,Cr:YSGG) was introduced in 1997 for the surgical needs of clinical dentistry in general practice. The erbium belongs to the rare earth which is embedded in a host crystal. The actual lasing process takes place in the Er ion Er^3+^. Two host crystals consisting of yttrium, aluminum, and garnet (Y_3_A_5_O_12_) and yttrium, scandium, gallium, and garnet (Y_3_Sc_2_Ga_3_O_12_) are added to the erbium. The interest to use these hard tissue laser in the treatment of soft tissue lesion was because of the properties of these lasers which are well-absorbed by chromophore water apart from hydroxy appetite crystals. Erbium laser energy is absorbed by collagen, hydroxyapatite, and water components. It allows the laser to cut soft tissue, tooth structure, and bone. In the noncontact mode, the incision is scalpel-like, with very little hemostasis. In contact mode, it performs soft tissue sculpting with adequate hemostasis. The Er,Cr:YSGG is the world’s most advanced dental laser, which is ideal all-tissue laser because all dental tissues contain water, for the multidisciplinary dentist who performs a broad spectrum of procedures. It delivers the highest level of clinician control, operating efficiency, flexibility in tip, and accessory selection. For optimal clinical results and patient comfort in hard and soft tissue procedures, the erbium lasers have set a new standard of clinical performance. The present case series aims to highlight the various soft tissue applications of Er,Cr:YSGG (Waterlase Biolase®, Biolase, Inc, San Clemente, California, USA) in pediatric patients.

**How to cite this article:**

Kumar G, Rehman F, Chaturvedy V. Soft Tissue Applications of Er,Cr:YSGG Laser in Pediatric Dentistry. Int J Clin Pediatr Dent 2017;10(2):188-192.

## INTRODUCTION

Many benign pathologies or oral anomalies that affect children’s soft tissues can be treated by dentists. Conventional treatment of these pathologies involves the use of the cold knife, cautery knife (electrocautery) or cryosurgery (using a gas expansion system or a cotton bud soaked in liquid nitrogen).^1^

Erbium, chromium-doped yttrium, scandium, gallium and garnet (Er,Cr:YSGG) was introduced in 1997 for the surgical needs of clinical dentistry in general practice. The erbium belongs to the rare earth which is embedded in a host crystal. The actual lasing process takes place in the Er ion Er^3^+. Two host crystals consisting of yttrium, aluminum and garnet (Y_3_A_5_O_12_) and yttrium, scandium, gallium and garnet (Y_3_Sc_2_Ga_3_O_12_) are added to the erbium.^2,3^ There is extensive literature on soft tissue management using lasers. Lasers have a number of advantages over conventional surgery for soft tissues as they reduce the amount of local analgesia required and the need for sutures is eliminated. Using lasers improves wound healing, which occurs faster and with less scarring than after conventional treatments. Healing is fastest after the application of erbium lasers, as they have a low thermal effect and reduce the requirement for antibiotics. The use of lasers for soft tissue problems is described herein by a series of case reports.

## CASE REPORTS

### Case 1: Mucocele Excision

**Figs 1A to F:**
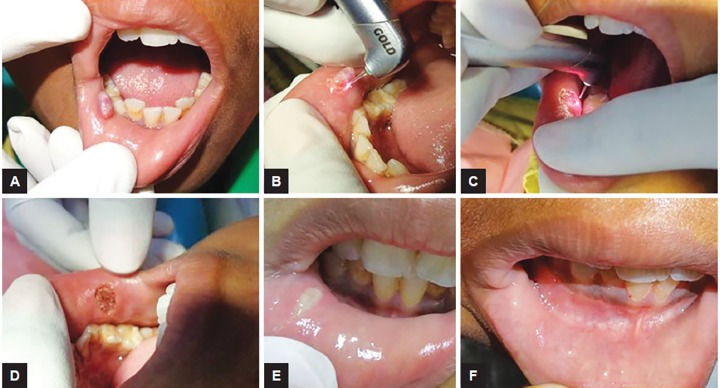
Mucocele excision: (A) Preoperative view showing mucocele location and size; (B) intraoperative view showing excision using Er,Cr:YSGG laser; (C) laser bandage being done; (D) immediate postoperative view; (E) follow-up 1 week postoperative; and (F) follow-up 6 months showing normal mucosa with no recurrence

A 9-year-old male child reported with the chief complaint of recurring swelling in lower lip for past 1 year. History revealed that the child had lip biting habit. The size of swelling often changes while having food. It was not associated with pain. Diagnosis of mucocele was made and laser excision was planned. Parameters of laser was adjusted according to soft tissue requirement, power-1.5 W, frequency-20 Hz, and air:water-60:40 for 10 seconds each time (Fig. 1).

### Case 2: Pyogenic Granuloma Excision

**Figs 2A to E:**
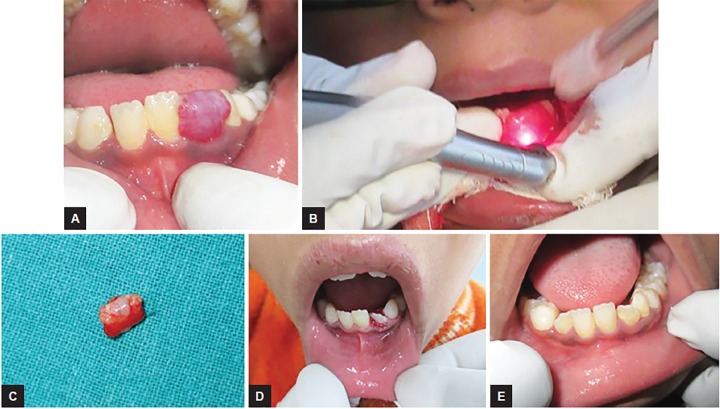
Pyogenic granuloma excision: (A) Preoperative view showing gingival overgrowth in relation to 32; (B) intraoperative view showing excision being done using Er,Cr:YSGG laser; (C) excised gingival tissue; (D) postoperative view exposing 32; and (E) follow-up 6 months

An 8-year-old male child showed gingival overgrowth in relation to left lower lateral incisor for past 4 months. On clinical evaluation it was associated with poor oral hygiene and profuse bleeding on probing. Overgrowth was planned to be excised with laser with power-1.5 W, frequency-20 Hz, and air:water-60:40 for 10 seconds each time till the tissue was completely detached from the tooth. Gingival tissue excised was sent for histopathology for confirmatory diagnosis and it came out to be pyogenic granuloma (Fig. 2).

### Case 3: Maxillary Frenectomy

**Figs 3A to E:**
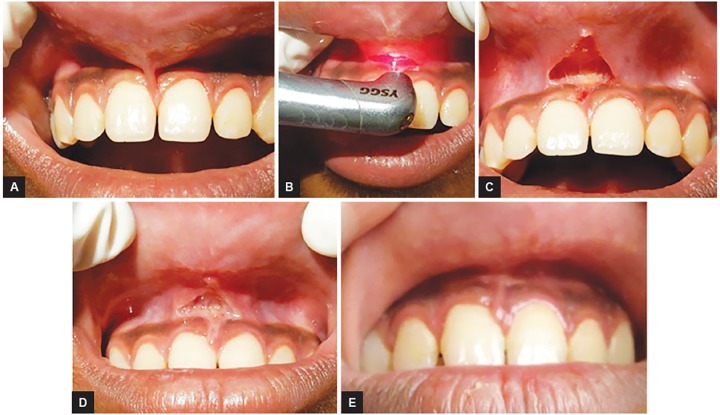
Maxillary frenectomy: (A) Preoperative view; (B) intraoperative view showing frenectomy being done using Er,Cr:YSGG laser; (C) immediate postoperative view; (D) follow-up 1 week; and (E) follow-up 1 year

A 12-year-old male patient reported with an unusual complaint of inability to clean the upper vestibule. On clinical evaluation, a high maxillary frenum was observed which was explained to the patient. Maxillary frenectomy was done using Er,Cr:YSGG laser with 1.75 W power, frequency-20 Hz, air:water-60:40 for 10 seconds each time till the fibrous band got detached with periosteum. Followed by laser bandage, wound healing took place by secondary intension with no complications. Only topical application of choline salicylate gel was prescribed during healing period (Fig. 3).

### Case 4: Gingival Fibroma Excision

**Figs 4A to C:**
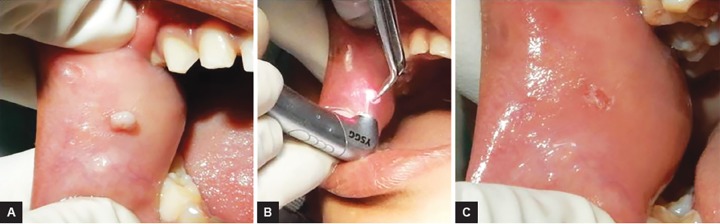
Gingival fibroma excision: (A) preoperative view; (B) intraoperative view; and (C) postoperative view

A 6-year-old girl reported with gingival growth on right buccal mucosa since 5 months. The child was anxious of surgery, so laser excision was planned under topical anesthesia. Tissue was excised within 5 seconds and child was very comfortable during the procedure. Excised tissue was sent for histopathological evaluation which confirmed it to be gingival fibroma (Fig. 4).

### Case 5: Exposure of Unerupted Teeth

**Figs 5A to C:**
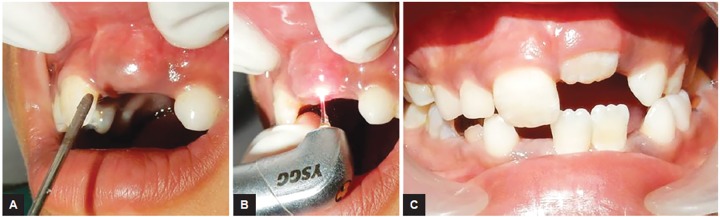
Exposure of unerupted teeth: (A) Preoperative view of unerupted 21 showing topical application of local anesthesia; (B) intraoperative view; and (C) follow-up 1 week showing eruption of 21

Mother of a 7-year-old child reported with the concern of non-eruption of upper front teeth. Even though we could wait for eruption at this stage of time, the concern of mother made us decide for exposing 21. Under topical anesthesia, only incision was given which resulted in eruption of 21 within 1 week’s time and mother was highly satisfied (Fig. 5).

### Case 6: Lingual Frenectomy

**Figs 6A to E:**
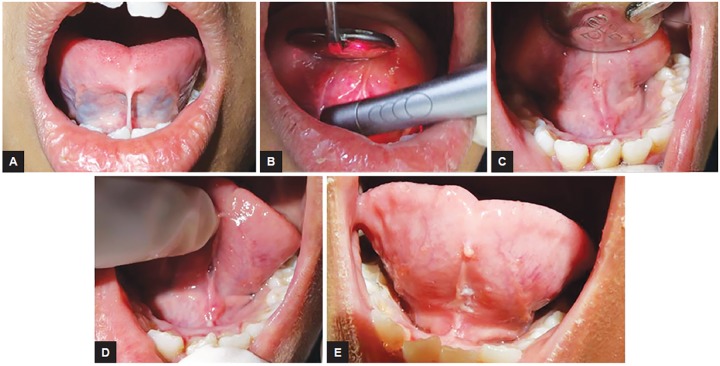
Lingual frenectomy: (A) Preoperative view; (B) intraoperative view; (C) immediate postoperative view; (D) follow-up 1 week; and (E) follow-up 1 month

A 1-year-old girl reported with tongue tie. Patient had impaired speech and restricted tongue movement. An intraoral examination revealed reduced mobility of the tongue due to a short lingual frenum. In maximum extension, the tongue did not reach the maxillary incisors and it had a bifid appearance. Lingual frenectomy was done with Er,Cr:YSGG laser with good postoperative healing and comfort. After one month, tongue mobility was normal. The maxillary incisors could now be reached by the tip of the tongue and the appearance was no longer bifid (Fig. 6).

## DISCUSSION

Currently, the use of Er,Cr:YSGG lasers in various fields of dentistry is gaining popularity due to its versatile nature. It can be used both for hard and soft tissue applications by simply altering the settings for individual procedure. The Waterlase™ Er,Cr:YSGG laser (Waterlase Biolase®, Biolase, Inc., San Clemente, California, USA) was used in the cases described in this paper. This has the advantage of utilizing either the preset parameters given for most of the procedures by the manufacturer itself or manual setting according to our requirement customized for every procedure as presented in the case reports here and in other papers published by Boj et al.^4-8^ The Er,Cr:YSGG laser is a hydrokinetic system based on photon liberation in an air-water spray, which causes strong explosions in the water droplets. The optical fiber delivery system ends in a sapphire crystal tip. The handpiece is similar to that of a conventional airotor handpiece, which facilitates behavior management in pediatric dentistry. This laser is especially useful in oral surgical procedures in children, as it involves a reduction in the amount of local analgesia and in the duration of intervention, the technique is easy and the laser produces a hemostatic effect that enhances visibility of the surgical area, which is a major advantage in children’s small mouths. Scarring is minimal (no tissue retraction). The laser eliminates the need for sutures and reduces postoperative edema, bleeding, infection, and pain and thus the use of medication.^5^ In this series, cases 4 and 5 were performed under topical anesthesia only without any discomfort to the child while other cases were performed under local infiltration. In a similar case report of exposure of unerupted central incisor, the child did not experience any pain even without the administration of injections or bleeding during the procedure and was immensely impressed with the excellent healing outcome.^9^ Also, the time taken to perform all these surgical procedures did not exceed 10 minutes each, rather, excision of fibroma and exposure of unerupted 21 took only a few seconds. No sutures were used in any of the presented cases. No postoperative complications were observed, healing was fast and uneventful. The same laser at a different output can be used to manipulate hard tissues. For labial frenectomies, Gontijo et al used a combination of a diode laser to manipulate soft tissues and the Er:YAG laser for the periosteum and the final collagen fibers.^10^

In the past, soft tissue surgical procedures were often rejected in children due to uncooperative behavior. It was thought that they could not be performed without general anesthetic.^11^ Several authors have stated that the use of lasers in soft and hard tissues causes less discomfort and is well-accepted by young patients and their parents. Thus, lasers can reduce psychological trauma and fear during the dental visit.^10,12^ As illustrated here in a series of 6 cases, the use of the Er,Cr:YSGG laser is ideal for soft tissues needing surgery in pediatric patients. Sarkar et al^13^ also reported four patients with different soft tissue lesions, such as peripheral ossifying fibroma, traumatic fibroma, intraoral lipoma, and gingival fibroma. No discomfort to the patient during and after laser procedure, no local anesthesia needed, no suture, and no antibiotics were prescribed. Minimal bleeding, no edema, and good wound healing were observed.

## CONCLUSION

The Er,Cr:YSGG laser can be used as alternative method to conventional scalpel method with the superiority of causing no discomfort and no complications with good wound healing in children.

## CLINICAL SIGNIFICANCE

The use of lasers in children is a boon as they aid in their behavior management. The inherent advantages of laserlike local analgesia, bloodless surgical procedures, and no discomfort during healing period make children more cooperative toward future dental treatment.

## References

[1] Taylor R, Shklar G, Roeber F (1965). The effects of laser radiation on teeth, dental pulp, and oral mucosa of experimental animals.. Oral Surg Oral Med Oral Pathol.

[2] Joseph SR (1997). The physics of surgical laser.. Oral Maxillofacial Surg Clin North Am.

[3] Coluzzi DJ (2000). An overview of laser wavelengths used in dentistry.. Dent Clin North Am.

[4] Boj JR, Hernandez M, Poirier C, Espasa E (2006). Treatment of pyo-genic granuloma with a laser-powered hydrokinetic system: case report.. J Oral Laser Appl.

[5] Boj JR, Hernandez M, Espasa E, Poirier C (2007). Laser treatment of an oral papilloma in the pediatric dental office: a case report.. Quintessence Int.

[6] Boj JR, Poirier C, Hernandez M, Espasa E (2007). Laser-assisted treatment of a dentigerous cyst: case report.. Pediatr Dent.

[7] Boj JR, Hernandez M, Espasa E, Poirier C, Espanya A (2008). Erbium laser treatment of an impacted first mandibular premolar: a case report.. J Clin Pediatr Dent.

[8] Boj JR, Poirier C, Espasa E, Hernandez M, Espanya A (2009). Lower lip mucocele treated with an erbium laser.. Pediatr Dent.

[9] Iyer VH (2014). Er,Cr:YSGG Laser as a treatment option for opercu-lectomy in children.. Int J Laser Dent.

[10] Gontijo I, Navarro RS, Haypek P, Ciamponi AL, Haddad AE (2005). The applications of diode and Er:YAG lasers in labial frenec-tomy in infant patients.. J Dent Child (Chic).

[11] Kotlow LA (2004). Lasers in pediatric dentistry.. Dent Clin North Am.

[12] Matsumoto K, Hossain M (2002). Frenectomy with the Nd:YAG laser: A clinical study.. J Oral Laser Appl.

[13] Sarkar S, Kailasam S, Iyer VH (2013). Effectiveness of Er,Cr:YSGG laser in the excision of different oral soft tissue lesions.. J Indian Acad Oral Med Radiol.

